# A Phosphoproteomic Analysis of Mycobacterial PknG-Mediated Host Immune Evasion

**DOI:** 10.1021/acs.jproteome.5c00416

**Published:** 2025-10-09

**Authors:** Seanantha S. Baros-Steyl, Kehilwe C. Nakedi, Tariq A. Ganief, Nelson C. Soares, Jonathan M. Blackburn

**Affiliations:** † Division of Chemical & Systems Biology, Department of Integrative Biomedical Sciences, Faculty of Health Sciences, 37716 University of Cape Town, Cape Town 7925, South Africa; ‡ Institute of Infectious Disease & Molecular Medicine, Faculty of Health Sciences, University of Cape Town, Cape Town 7925, South Africa; § Center for Applied Translation Genomics, Mohammed Bin Rashid University of Medicine and Health Sciences, Dubai Health, Dubai 505055, United Emirates; ∥ College of Medicine, Mohammed Bin Rashid University of Medicine and Health Sciences, Dubai Health, Dubai 505055, United Arab Emirates; ⊥ Laboratory of Proteomics, Department of Human Genetics, National Institute of Health Doutor Ricardo Jorge, Lisbon 1649-016, Portugal; # Comprehensive Health Research Centre , NOVA Medical School, University NOVA of Lisbon, Lisbon 1169-056, Portugal

**Keywords:** mass spectrometry, phosphoproteomics, post-translational modifications, Mycobacterium tuberculosis, phosphorylation, serine/threonine protein kinases

## Abstract

Pathogenic mycobacteria, such as *Mycobacterium tuberculosis*, modulate the host immune system to evade clearance and promote long-term persistence, leading to disease progression or latent infection. Understanding how these mycobacteria evade elimination is key to uncovering the molecular mechanisms of infection. Protein kinase G (PknG) in pathogenic mycobacteria plays a critical role in avoiding macrophage clearance by inhibiting phagosome-lysosome fusion; however, the exact mechanism is not completely understood. To investigate the role of PknG during early events of macrophage infection, RAW 264.7 macrophages were infected with *Mycobacterium bovis* BCG wild-type and PknG knockout mutant strains. Phosphoproteomic analysis, including TiO_2_-based phosphopeptide enrichment and LC–MS/MS, identified 3003 phosphosites across 1638 host proteins. Differential expression analysis revealed 143 phosphosites significantly altered between wild-type and mutant infections, with 95 exhibiting increased phosphorylation in the presence of PknG. Additionally, 34 phosphosites were exclusively phosphorylated in the presence of PknG. Functional analysis demonstrated that PknG kinase activity reprograms normal macrophage function by interfering with host cytoskeletal organization, phagosome maturation, and programmed cell death, establishing a new role for PknG in directing the fate of mycobacteria within macrophages. Differentially phosphorylated proteins in this study serve as a foundation for further validation and the assignment of PknG host substrate assignment.

## Graphical abstract

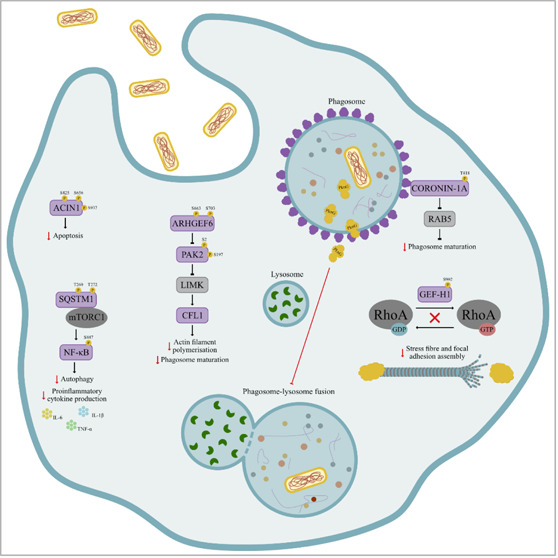

## Introduction

Tuberculosis (TB) remains one of the leading causes of death from infection worldwide.^1^ The high virulence of *Mycobacterium tuberculosis* (*Mtb*) is partly due to its ability to survive and replicate within alveolar macrophages, establishing reservoirs of live bacteria that promote the persistence and recurrence of the disease. An estimated one-quarter of the global population harbors a latent TB infection (LTBI),^2^ which, although asymptomatic, represents a reservoir for the potential reactivation and transmission of TB.^3^

Pathogenic mycobacteria suppress various host processes, including phagosome-lysosome fusion, programmed cell death, and antigen presentation.^4^ In addition, *Mtb* promotes the activation of pathways involving mitogen-activated protein kinases (MAPKs), calcium signaling, and interferon-γ (IFN-γ) to weaken mycobactericidal responses.^5,6^ Mycobacterial Ser/Thr protein kinases and phosphatases are known to play a role in interfering with host-signaling pathways during infection, thereby promoting intracellular survival.^7–10^

The mycobacterial secretory protein kinase G (PknG) is a known virulence factor and contributes to the inhibition of phagosome-lysosomal fusion during early stage infection.^9^ This is presumably the result of PknG secretion and, thus, a kinase-mediated signaling mechanism through which PknG interacts/phosphorylates host protein substrates, thereby facilitating intracellular survival of the pathogen.^9,11^ Experimental evidence shows that inactivation of PknG by gene disruption or chemical inhibition results in lysosomal localization and mycobacterial cell death in infected macrophages.^9,11^ Although these processes seemingly depend on the kinase activity of PknG, the exact molecular basis by which PknG enables the establishment of a niche inside host macrophages remains unclear. Thus, understanding the mechanistic connection between PknG in such complex regulatory signaling networks and mycobacterial survival in macrophages requires a detailed view of the phosphorylation events occurring during infection.

Our previous in vivo study identified novel mycobacterial physiological substrates for PknG by comparing the phosphoproteome dynamics during growth in liquid culture of *Mycobacterium bovis* BCG wild-type (WT) against the respective PknG gene knockout (BCG-Δ*pknG*) mutant.^12^ The study revealed a new set of mycobacterial protein targets that were exclusively phosphorylated in *M. bovis* BCG-WT and not in the BCG-Δ*pknG* mutant. Further validation of these initial results using PRMs and docking analyses allowed the identification of a set of 23 mycobacterial proteins as novel physiological substrates for PknG.^12^

Here, by employing a similar strategy, we achieved an expanded understanding of mycobacterial Ser/Thr/Tyr phosphorylation networks within macrophages infected by *M. bovis* BCG. Recognizing that the initial stages of host cell infection by pathogens are pivotal in dictating the trajectory and outcome of the infection, the investigation focused on the host–pathogen interactions during this early stage of infection at the post-translational level. This was achieved through a comparative analysis of phosphoproteomic alterations in RAW 264.7 macrophage cells postinfection with either *M. bovis* BCG-WT or the BCG-Δ*pknG* mutant. The study identifies probable protein phosphorylation mechanisms by which pathogenic mycobacterial PknG reprograms macrophage function to promote their survival and persistence in macrophages.

## Experimental Procedures

### *M. bovis* BCG Strains and Bacterial Culture

Three bacterial strains were used in this study: *M. bovis* GFP-labeled BCG-WT, BCG-Δ*pknG* (PknG gene knockout), and BCG-WT (BCG-Δ*pknG* complement). The BCG-Δ*pknG* and BCG-WT strains were generated as described in Walburger et al. (2004)^9^ and kindly donated by Professor Jean Pieters from the University of Basel, Switzerland. All strains were propagated in 7H9Middlebrook broth (BD Difco) supplemented with 10% OADC (BD BBL), 0.05% Tween 80, and 0.2% (v/v) glycerol. Cultures were maintained at 37 °C with continuous agitation at 120 rpm. Growth progression was monitored by daily measurements of the optical density (OD_600_) until the logarithmic phase (OD_600_ = 0.8) was reached.

### Culture Conditions of RAW 264.7 Macrophages

Our initial cell line stocks were kindly donated by Dr T. Heunis from the University of Stellenbosch, South Africa. Frozen cell line stocks were defrosted at 37 °C in a water bath. The cells in suspension were pelleted by centrifugation at 800*g* for 3 min, followed by two rounds of washing with prewarmed PBS and repelleting. The resulting pellets were resuspended in Dulbecco’s modified Eagle medium (Difco), supplemented with pyruvate and glutamine (Difco) and 10% (v/v) heat-inactivated fetal calf serum (Sigma-Aldrich) - referred to as D10. The cells were seeded into T25 tissue culture flasks (Lasec) at 30% confluence and grown in a humidified (95% humidity) incubator at 37 °C with 5% CO_2_. Cells from passages 10 (P10) to 13 (P13) were used.

### Infection of RAW 264.7 Macrophages with Mycobacterial Strains

Macrophage cell lines were grown until a confluency (∼70%) was reached. An extra flask was prepared for counting before infection. To prepare the infection buffer, mycobacterial cultures at logarithmic stage were washed twice with PBS and the cell pellet was resuspended in D10. Bacterial clumps were minimized using a water bath sonicator at a power setting 4 for 10 min. Bacterial cells were then passed through a 22G needle ten times using a 10 mL syringe, followed by gentle centrifugation (100*g* for 5 min) to sediment clumps. The supernatant was removed, and the cultures were diluted in D10 to the desired OD_600_ based on the target multiplicity of infection (MOI). Macrophage media was aspirated, and the cells were washed twice with prewarmed PBS. Subsequently, they were incubated with the infection buffer at an MOI of 4. Following a 30 min incubation period to facilitate uptake, the cells underwent five washes with PBS. Fresh D10 medium was then introduced, and the cells were further incubated for 30 min before being harvested. For the uninfected controls, the macrophage medium was aspirated, and the cells were washed twice with prewarmed PBS before incubation with D10 medium as above.

Aliquots derived from macrophage cell culture supernatants, postinfection at 30 and 60 min, were also harvested. This involved centrifugation at 1000*g* for 10 min at 4 °C to remove any cells or cellular debris. Following this, the clarified supernatants were flash-frozen using liquid nitrogen and stored at −80 °C until the samples were required for analysis using Olink proximity extension assays (PEA).

### Protein Extraction and Quantification

Cells were lysed with a modified RIPA buffer [comprising 50 mM Tris–HCl at pH 7.4, 150 mM NaCl, 1 mM EDTA, 0.1% sodium deoxycholate, and 1% (w/v) SDS] supplemented with 1X protease and 1X phosphatase inhibitor cocktail tablets (Roche). The mixture was gently agitated on ice for 5 min prior to treatment with endonuclease benzonase (Sigma-Aldrich). Protein precipitation was carried out by using a chloroform/methanol precipitation method. The resulting protein precipitates were subsequently resolubilized in denaturation buffer (6 M urea and 2 M thiourea in 50 mM Tris–HCl, pH 8.5). Protein concentrations were determined using a modified Bradford assay.^13^

### Colony Forming Unit (CFU) Assay

For the Colony Forming Unit (CFU) assay, lysates of the infected cells were subjected to a 10-fold dilution. Subsequently, 100 μL of each dilution was plated in triplicate on Middlebrook 7H10 agar plates (BD Difco). These plates were then incubated at 37 °C with 5% CO_2_ to facilitate the growth of internalized mycobacteria. After 3–4 weeks, CFUs were manually counted.

### Tryptic Digestion and Phosphopeptide Enrichment

In-solution tryptic digestion was carried out on 550 μg of total protein. Initially, proteins were reduced with 1 mM dithiothreitol (DTT) at room temperature (RT) with gentle agitation for 1 h. Alkylation was then carried out with 5.5 mM iodoacetamide (IAA) at RT for 1 h in the dark, preventing disulfide bond formation. Predigestion with Lys-C endopeptidase (Wako) occurred for 3 h at 30 °C, followed by a 4-fold dilution with HPLC-grade water to a final urea concentration of ∼1.5 M. The diluted sample was then digested overnight with trypsin (1:100 ratio) at 30 °C. The digestion was quenched with 1% trifluoroacetic acid (TFA) (Sigma-Aldrich) to a final concentration of 0.1%.

Digested peptides were desalted using in-house reverse-phase C18 chromatography (Millipore) in preparation for mass spectrometry (MS) analysis. Briefly, C18 columns were equilibrated twice with solvent B [80% acetonitrile (ACN) and 0.1% formic acid (FA)] and centrifuged at 3000*g* for 30 s. Samples underwent two washes with solvent A (2% ACN and 0.1% FA) before being loaded onto the column. Peptides were eluted with solvent C (60% ACN and 0.1% FA) and dried at RT in a SpeedVac vacuum concentrator (Savant). Out of the total digested protein, 50 μg was reserved for proteome analysis, while the remaining 500 μg was enriched for phosphorylated peptides using a TiO_2_ phosphopeptide enrichment kit (Thermo Fischer Scientific), following the manufacturer’s instructions.

### Liquid Chromatography with Tandem Mass Spectrometry (LC–MS/MS) Analysis

LC–MS/MS analysis was conducted using the Dionex Ultimate 3500 RSLC Nano System (Thermo Fisher Scientific) coupled to a Q Exactive mass spectrometer (Thermo Fisher Scientific). Following desalting as previously described, both proteome and phosphoproteome peptides were resuspended in 15 μL of solvent A. From this, 4 μL was loaded at 1 μg per injection into the LC–MS/MS system. The total ion current was used as a proxy to ensure a consistent peptide loading.

Peptides were chromatographically separated using a 75 μm internal diameter, 25 cm column, packed in-house with a reversed-phase 3 μm Kinetex core–shell C18 resin (Phenomenex). The flow rate was set at 400 nL/min, and the temperature was maintained at 40 °C. The gradient profile consisted of 2% solvent B (0.1% FA, ACN) increased to 8% solvent B over 2 min, followed by increasing to 23% solvent B over 80 min. This was followed by a washout at 80% solvent B for 10 min.

MS1 spectra were acquired between 300 and 1750 Thompson at a resolution of 75000 with an AGC target of 3 × 10^6^ within 250 ms. Using a dynamic exclusion window of 90 s, the top 10 most intense ions were selected for higher-energy collisional dissociation fragmentation with a normalized collision energy of 28. MS2 spectra were acquired at a resolution of 17500 with a minimum AGC target of 1 × 10^3^, acquired within 80 ms.

### Protein and Phosphopeptide Identification

Raw data files were processed using MaxQuant version 2.0.3.0. MS/MS spectra were matched against the *Mus musculus* and *M. bovis* (strain BCG/Pasteur 1173P2) reference proteomes downloaded on 27 April 2023 from UniProt (https://www.uniprot.org). The MaxQuant built-in Andromeda search algorithm^14^ was employed to align spectra with the reference proteome, with mass tolerances set at 20 ppm for precursor ions and 7 ppm for fragment ions. The “match-between-runs” functionality was enabled, allowing peptide identifications to be transferred between raw files based on a matching time window of 0.7 min and an alignment time window of 20 min, focused on precise retention time and mass-to-charge ratio (*m*/*z*). False discovery rates (FDRs) for identifications were estimated by using a target-decoy database. Carbamidomethylation of cysteine residues was specified as a fixed modification across all groups. Variable modifications considered were oxidation of methionine and protein N-terminal acetylation for the proteome files with the addition of phosphorylation of serine, threonine, and tyrosine (Ser/Thr/Tyr) residues for the phosphoproteome files. Trypsin and Lys-C were selected as digestion enzymes with an allowance for up to two missed cleavages.

### Olink PEA

To delineate the early immunological and inflammatory profiles elicited by mycobacterial infection, the Olink Target 96 Inflammation Panel (Olink Proteomics, Uppsala, Sweden) was employed, according to the manufacturer’s instructions. Frozen supernatants were thawed on ice, randomized, and transferred to a 96-well plate prior to running the assay. The detection threshold for each protein biomarker was established based on the mean value derived from triplicate negative controls. Any measurement falling below this limit of detection was excluded from subsequent analyses. The assay’s output is quantified in terms of Normalized Protein eXpression (NPX), an arbitrary log2-scale scale unit.

### Statistical Processing and Bioinformatics Analysis

The Proteus package^15^ in R was used for evidence data exploration, quality checks, and visualization at the peptide level. Perseus version 1.6.14.0 software^16^ was used for quality control, statistical processing, and protein annotation. Protein identifications were filtered for those identified by site, reverse database hits, and potential contaminants. The log2-transformed protein abundance values were then filtered to contain at least three valid values among the four biological replicates per condition. An ANOVA with a Benjamini-Hochberg (BH) FDR threshold of 0.05 was performed to detect differentially abundant proteins.

Phosphopeptide identifications were filtered for reverse hits, potential contaminants, and a localization probability >0.75. The phosphoproteomic intensity values were log2-transformed before normalization to the proteomic intensities by subtraction to account for protein abundance differences. The normalized intensities were then filtered to contain a minimum of three valid values among the four biological replicates per condition. A median normalization was also performed across the data set, followed by a two-sample *t*-test with a BH FDR threshold of 0.05. The identified differentially phosphorylated peptides were functionally annotated and filtered for unique entries. In addition, a presence/absence analysis was performed on the quality-controlled data set by filtering for phosphopeptide identifications that were present in at least three of the four replicates of BCG-WT and absent in all four replicates of the BCG-Δ*pknG* mutant.

The Olink NPX data was analyzed using a custom-developed R script designed to systematically identify and exclude outlier samples, perform an ANOVA, and visualize the differentially abundant immunological and inflammatory markers. Benjamini-Hochberg *posthoc* correction was used to control the FDR. Adjusted p-values <0.05 were considered statistically significant.

### Functional Enrichment and Network Analysis

To identify pathways altered by PknG during mycobacterial infection, the complement of differentially abundant proteins and phosphoproteins was assessed for enriched Kyoto Encyclopaedia of Genes and Genomes (KEGG) pathways using the enrichment analysis tool of the stringApp plug-in within Cytoscape^17,18^ with FDR <0.01. The *M. musculus* genome was used as the background. Functional enrichment is based on over-representation analysis using hypergeometric tests. Similarly, the complement of differentially phosphorylated proteins and the subset of phosphoproteins displaying increased phosphorylation levels were used to retrieve enriched Gene Ontology (GO) terms using the stringApp. The enrichment results were filtered for redundancy with a cutoff of 0.25, and GO terms and KEGG pathways identified with less than three proteins were ignored.

The functional enrichment networks were visualized using the clusterProfiler package.^19^ Briefly, the genome-wide annotation library (org.Mm.eg.db) for *M. musculus* was downloaded from Bioconductor (www.bioconductor.org). UniProt IDs of the differentially phosphorylated proteins were converted to Entrez Gene IDs using org.Mm.eg.db as the organism database. A 90.28% mapping rate of our proteins to the annotation database was achieved. Gene-concept networks were generated using the GO enrichment analysis results.

### Phosphorylation Motif Analysis

In silico motif analyses were conducted on peptides that exhibited significant Ser and Thr phosphorylation in the presence of PknG using the pLogo visualization tool.^20^ The analysis was designed to identify phosphorylation site motifs, employing a foreground data set comprised of phosphopeptide sequence windows that demonstrated increased abundance. To ensure the statistical robustness of the motifs identified, a significance threshold was set at a *p*-value ≤0.05, with the application of a Bonferroni to robustly minimize false positives in motif identification. The global observed phosphoproteome or the *M. musculus* proteome were used as background data sets. This comparative approach allows for the discernment of phosphorylation patterns over-represented in the context of PknG activity relative to the general protein expression profile in mice.

### Confocal Imaging

For fixed cell imaging experiments, glass coverslips were first sterilized and then coated with poly-l-lysine (Sigma-Aldrich) before being placed into 12-well plates. RAW 264.7 macrophages were seeded at a density of 0.1 × 10^6^ cells and cultured until they reached 70% confluence. The macrophages were then infected with GFP-labeled BCG-WT at an MOI of 4, as described above. Postinfection, the cells were washed, fixed using 4% paraformaldehyde for 10 min, and permeabilized with 0.1% Triton X-100 (Sigma-Aldrich) for 5 min. Following thorough washing, the actin cytoskeleton was stained with a Phalloidin-Atto 565 (Sigma-Aldrich). Fixed-cell images were captured as z-stacks using a Zeiss 880 LSM confocal microscope with a C-Apochromat 40*x*/1.2 W Corr, UV–vis-IR objective (Zeiss). Mycobacteria channel (GFP) was obtained using excitation (*E*_x_): 488 nm; emission (Em): 520 nm. Actin cytoskeleton channel (Phalloidin-Atto 565) was obtained using *E*_x_: 562 nm and *E*_m_: 590 nm. Fluorescent and bright-field signals were acquired sequentially. At least three fields were imaged for each well.

RAW 264.7 macrophages were seeded for live-cell imaging at a density of 0.1 × 10^6^ in a four-chamber, 35 mm diameter, 14 mm glass bottom imaging dish with a No. 1.5 coverslip (MatTek Corporation). The cells were grown until they reached 70% confluence and then infected with GFP-labeled BCG-WT at an MOI of 4, as described above. Time-lapse imaging was performed using a Zeiss 880 LSM confocal microscope with a C Plan-Apochromat 63*x*/1.4 Oil, DIC UV–vis-IR M27 objective (Zeiss). Image acquisition was initiated 8 min postinfection.

All images were saved in “.czi” format to preserve data integrity and were processed using Fiji software (version 2.15.0). Confocal z-stacks were converted into average intensity projections and visualized as montages. Time-lapse images were embedded with timestamps to provide temporal context and saved as videos.

## Results

### Internalization of Mycobacteria by RAW 264.7 Macrophages

To capture early phosphoproteomic events, our infection assays were incubated for a 30 min uptake, followed by a 30 min chase to allow for the activation of correlated pathways. Previous studies have established the use of low MOIs for in vitro studies of *M. bovis* BCG macrophage infections.^24,25^ Chávez-Galán et al. (2016) showed that RAW264.7 macrophage infected with BCG at an MOI of 1 activated higher and sustained levels of proinflammatory cytokines and transcription factors, while an MOI of 0.1 was more efficient for early stimulation of IL-1β, MCP-1, and KC.^24^ Thus, BCG infection at low dose represents an efficient in vitro model to study macrophage-BCG interactions under conditions that maintain macrophage viability while also better mimicking likely physiological infection conditions. Therefore, an MOI of 4 was chosen in this study to mitigate the dominance of cell death signals in the resultant proteomic and phosphoproteomic data.

Confocal microscopy provided visual evidence of mycobacterial internalization under the experimental conditions (Figure 1). To substantiate this observation, live-cell imaging was conducted, providing a detailed temporal analysis of *M. bovis* BCG internalization by macrophages (see Videos S1, S2, S3, S4, S5, S6, S7 and S8). This enabled the confirmation of *M. bovis* BCG uptake both before and after a 30 min incubation period. This observation aligns with the robust identification of 87 *M. bovis* BCG proteins in the wild-type BCG infection proteomic data set, constituting ∼2.8% of the total observed protein mass. Twenty five of these proteins were identified by at least two peptides and were also consistently observed in at least three out of four replicates in both infection conditions (Table S1). Notably, the majority of these 25 *M. bovis* BCG proteins, while associated with the cell wall, are considered cytoplasmic (e.g., GroEL-GroES complex, 50S and 30S ribosomal proteins, and other chaperones) and rank among the top 50 most abundant *M. bovis* BCG proteins in in vitro cultured cells.^26^ We did not observe PknG or PknB, nor or their reported mycobacterial substrate GarA,^32,35^ among the mycobacterial proteins, presumably due to their lower expression levels coupled with MS dynamic range considerations in the background of a murine macrophage proteome.

**Figure 1:**
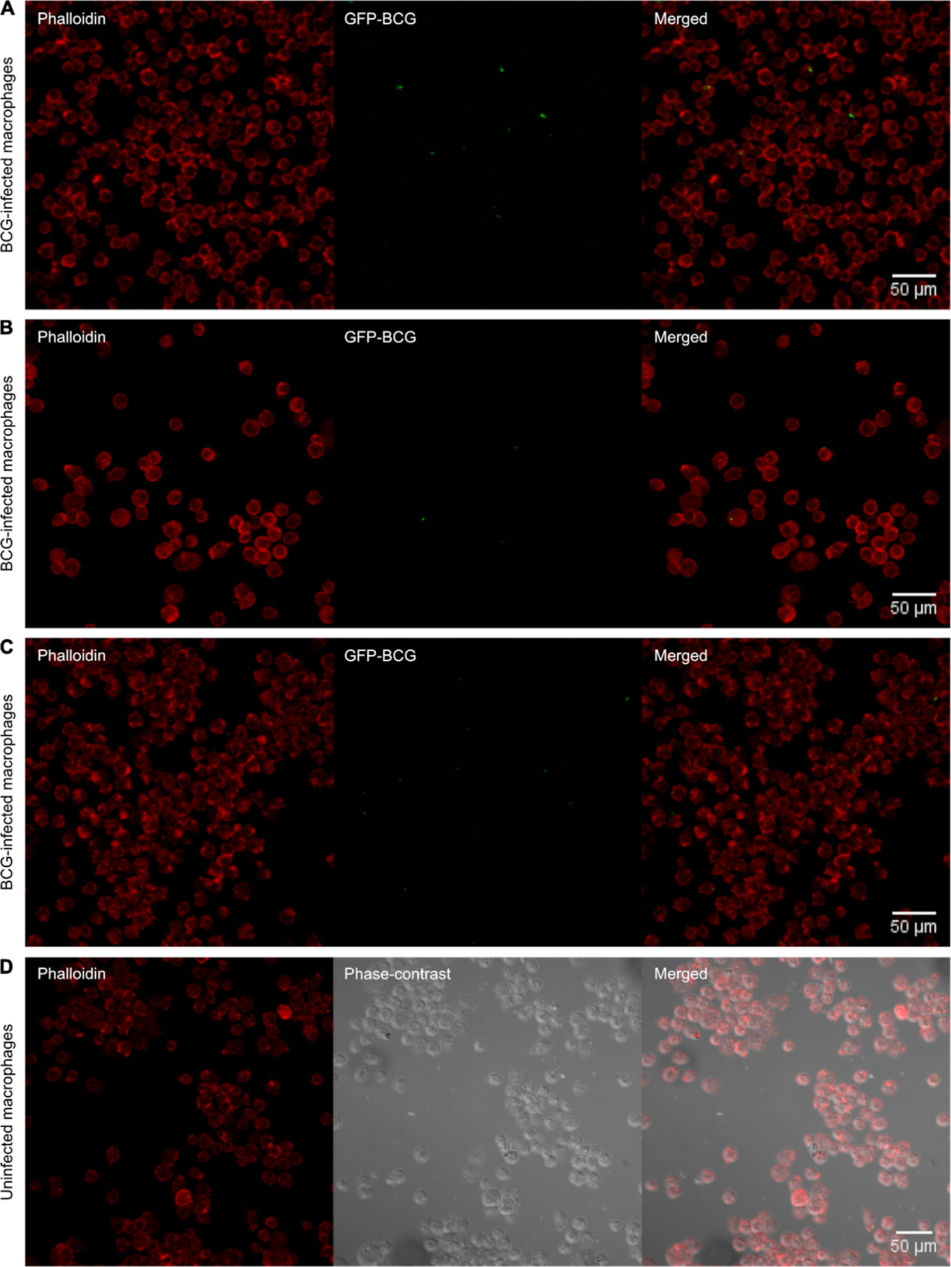
Confocal microscopy average intensity projections of RAW 267.4 macrophages infected at an MOI of 4 with GFP-expressing *M. bovis*BCG, observed 30 min postinfection. (A–C) BCG-infected macrophages and (D) uninfected controls. The macrophages are stained with Phalloidin (red) to highlight the actin cytoskeleton, while the *M. bovis* BCG is visualized through GFP fluorescence (green), demonstrating the presence of internalized bacilli within macrophages at 30 min postinfection. Scale bar: 50 μm.

### Intracellular Survival of Mycobacteria in RAW 264.7 Macrophages

The survival dynamics of *M. bovis* BCG-WT compared to the BCG-Δ*pknG* mutant were monitored by measuring CFU counts at 5 h and 24 h intervals postinfection at an MOI of 4 (Figure 2). At the initial time point, macrophages displayed a higher uptake of the BCG-Δ*pknG* mutant relative to that of the WT, suggesting increased early interaction with the mutant strain. Over 24 h postinfection, the BCG-WT strain exhibited a 2-fold increase in CFU, whereas the growth of the null mutant was constrained by the macrophages, consistent with findings by Walhburger et al. (2004).^9^ It is pertinent to note that BCG does not exhibit significant propagation in D10 media within 24 h in the absence of macrophages.

**Figure 2:**
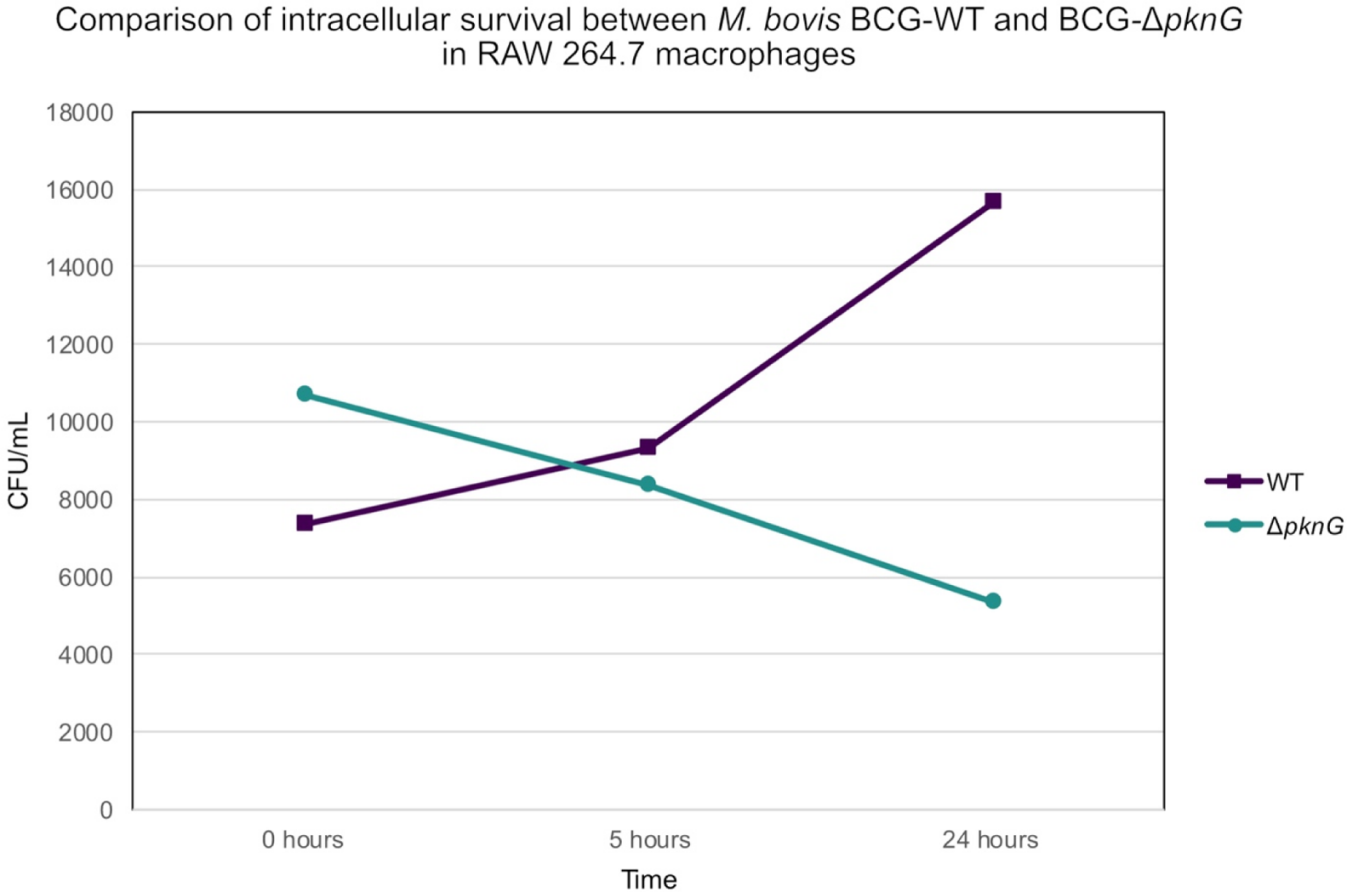
Survival of *M. bovis* BCG-WT and BCG-Δ*pknG* mutant in macrophages at different time points postinfection. Histogram showing the bacterial growth of the different mycobacterial strains in RAW 264.7 macrophages at 0, 5, and 24 h postinfection.

### Identification of PknG-Responsive Quantitative Proteomic and Phosphoproteomic Changes in the RAW 264.7 Macrophages during Infection

To decipher the host mechanisms exploited by PknG to promote the pathogenesis of TB, a label-free quantitative phosphoproteomic approach was employed to examine the global phosphorylation dynamics associated with PknG during *M. bovis* BCG macrophage infection. Various studies have demonstrated such methodology to be a high-throughput platform for identifying targets of protein kinases.^12,27–29^ Proteomic experiments were run in parallel with the phosphoproteomic workflow (Figure 3). Four biological replicates were analyzed for each condition to increase the phosphoproteome coverage. The evidence data was used to assess the quality of peptide identifications.

**Figure 3:**
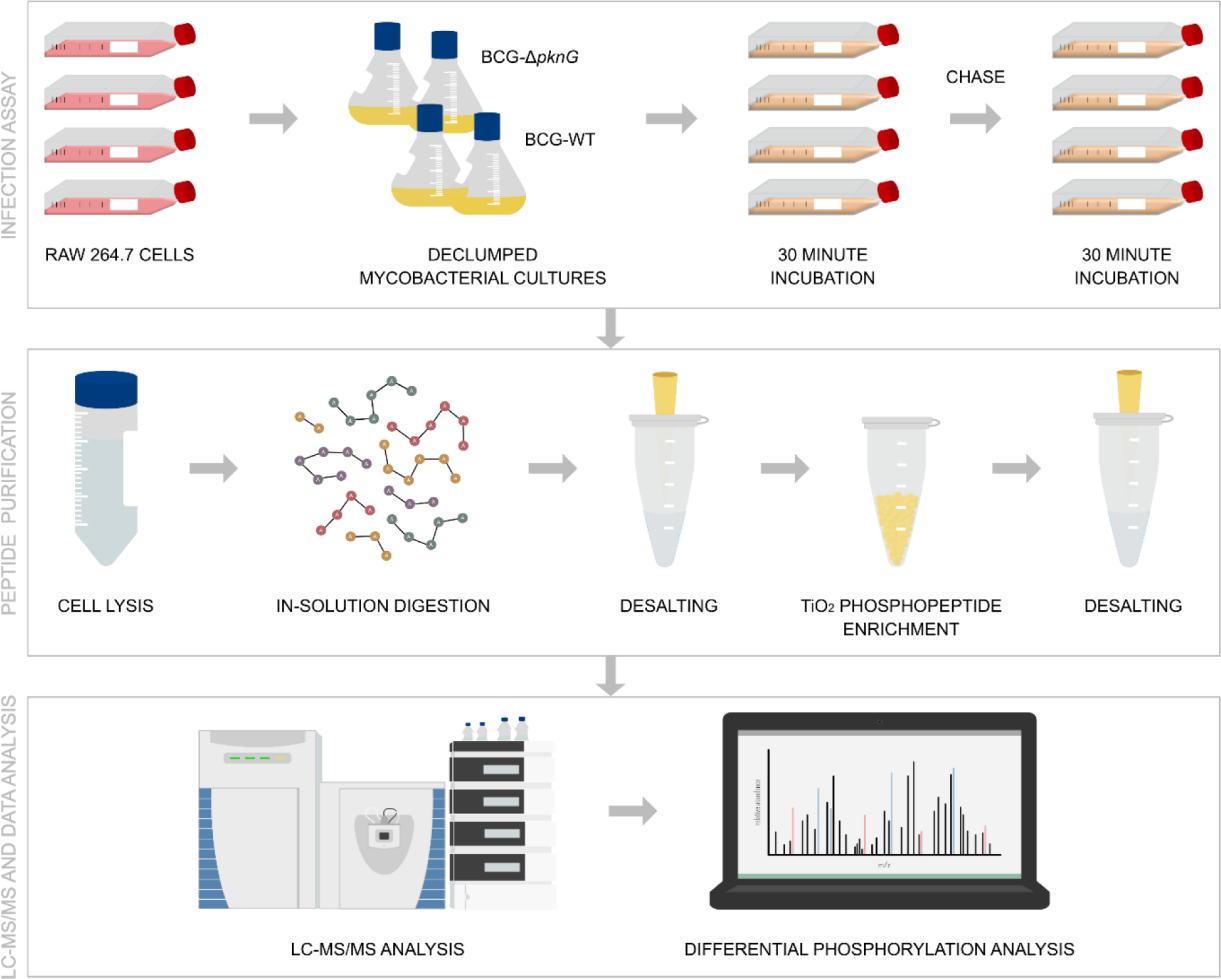
Proteomic and phosphoproteomic workflow used in this study. *M. bovis* BCG-WT and BCG-Δ*pknG* strains were grown until mid log phase and RAW 264.7 cells were cultured in D10 media until confluent. Infection assays were carried out at an MOI of 4 with a 1 h incubation. The RAW 264.7 cells were washed and lysed in the presence of protease and phosphatase inhibitors, followed by in-solution digestion with Lys-C and Trypsin. The samples were purified using reverse-phase C18 chromatography and an aliquot reserved for proteome analysis. Phosphopeptides were enriched using a TiO_2_ phosphopeptide enrichment kit, followed by another cleanup step. LC–MS/MS data acquisition was then performed on an HPLC coupled to a Q Exactive mass spectrometer. The raw data files were processed using MaxQuant and analyzed using Perseus, R, and other software packages.

Figure 4A–D illustrates the detailed visualizations generated to assess the quality and distribution of the peptide data, ensuring the reliability of our subsequent analyses. The hierarchical clustering (Figure 4C) and principal component analysis (Figure 4D) demonstrate appropriate sample grouping based on proteomic profiles, with the uninfected controls (UN) clustering distinctly from both the BCG-WT (WT) and BCG-Δ*pknG* (KO) infection conditions. This clear separation reinforces the validity of the experimental setup and suggests effective infection following the 30 min uptake, as indicated by the distinct proteomic signatures of the infected samples relative to the uninfected controls. These results strengthen the confidence in the infection model and support reliable downstream analyses of infection-induced changes.

**Figure 4:**
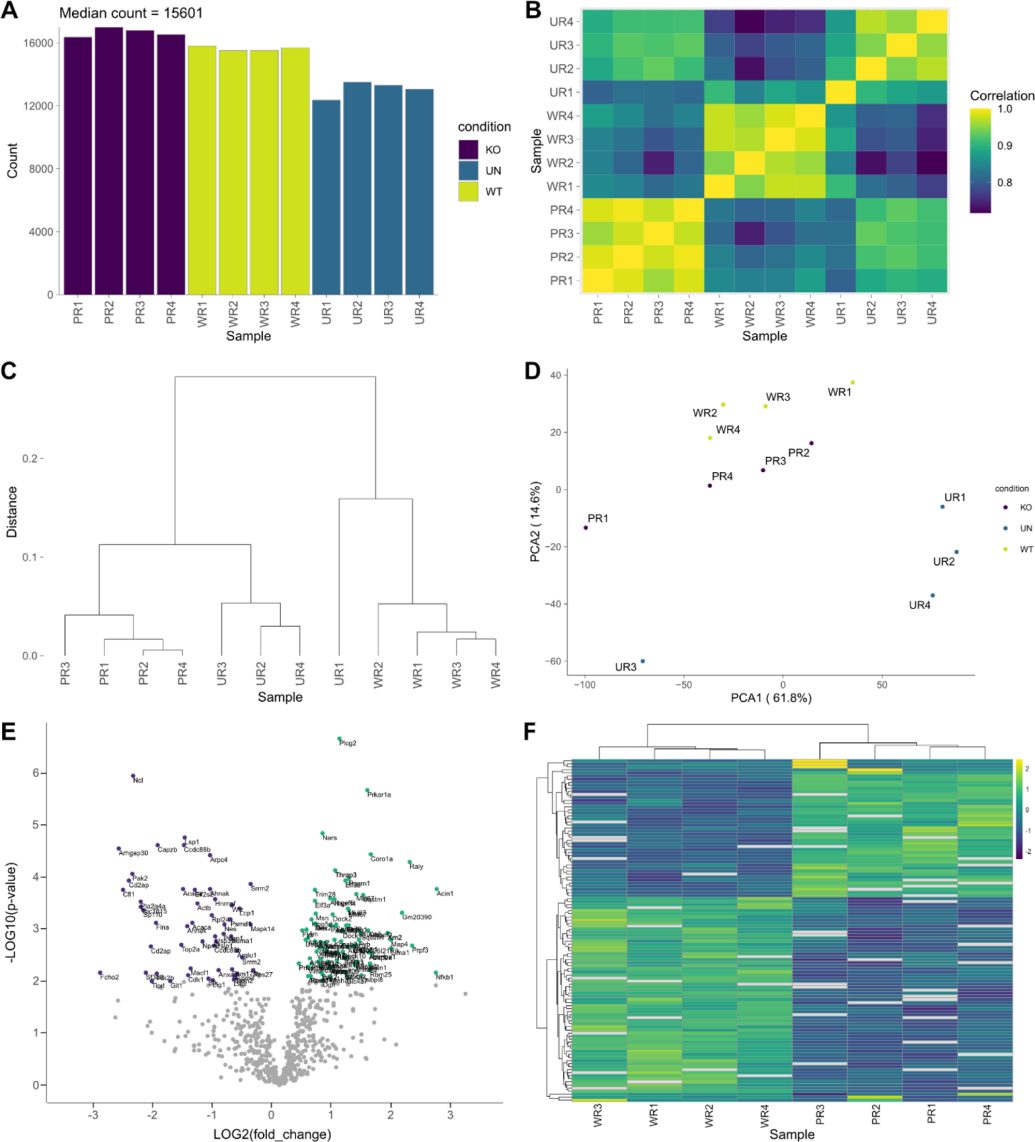
Comprehensive peptide and phosphopeptide analysis. (A) Distribution of peptide counts across samples. (B) Distance matrix plot displaying Pearson’s correlation coefficients, providing a pairwise comparison of sample similarity based on peptide intensities. (C) Dendrogram representing hierarchical clustering of samples based on peptide identifications. (D) Principal-component analysis of samples based on peptide identifications. (E) Volcano plot highlighting the significantly differentially phosphorylated peptides. Scatter plot showing the phosphopeptide log2 fold-change (WT/BCG-Δ*pknG*) plotted against the -log10­(p-value) highlighting the differentially phosphorylated peptides (two-sample *t*-test with BH FDR <0.05). Phosphopeptides with decreased and increased abundance in the presence of PknG are indicated in purple and green, respectively. (F) Hierarchical clustering analysis of phosphopeptides with significantly increased abundance in the presence of PknG. Heatmap showing the abundance levels and clustering of phosphopeptides with notably higher abundance levels (two-sample *t*-test with BH FDR <0.05; log2FC ≥ 1 WT/BCG-Δ*pknG*). Phosphopeptide intensities exhibiting increased abundance are colored green, while those with decreased abundance are colored purple.

The proteomic analysis identified 3785 protein groups after the initial filtering. The log2-transformed label-free quantification protein abundance values were then filtered to contain a minimum of three valid values in each group, resulting in 1278 protein groups remaining. After differential analysis, 542 proteins were considered to have significantly different abundance levels between the *M. bovis* BCG-WT and BCG-Δ*pknG* samples (Table S2). Of these, 497 host proteins exhibited increased abundance (Table S3).

A total of 3003 phosphosites (p-sites) were identified. After normalization to the proteome and valid value filtering, 789 highly confident phosphopeptides mapped to 422 phosphoproteins were taken for further analysis. The distribution of phosphoserine (pS), phosphothreonine (pT), and phosphotyrosine (pY) sites was 85.3%, 13.4%, and 1.3%, respectively. Moreover, the majority of the peptides were singly phosphorylated (95.1%), whereas approximately 4.9% were multiply phosphorylated. Figure S1 illustrates the relative fractions of pS, pT, and pY observed among the differential and nondifferential p-site, peptide, and protein identifications. The MS proteomics and phosphoproteomics data have been deposited to the ProteomeXchange Consortium (http://proteomecentral.proteomexchange.org) via the PRIDE partner repository with the data set identifier PXD031055.^30,31^

The normalized log2-transformed intensities of the RAW 264.7 cells infected with *M. bovis* BCG-WT and BCG-Δ*pknG* mutant were compared using a two-sample *t*-test with a BH FDR <0.05. A total of 149 peptides were significantly differentially phosphorylated (Table S4). Of these, 95 p-sites showed significantly increased abundance in the presence of mycobacterial PknG (Table S5), with 44 phosphoproteins presenting notably elevated abundance levels with log2FC > 1 (Table 1). A volcano plot (Figure 4E) revealed significantly differentially phosphorylated peptides with FDR <0.05. Hierarchical clustering of the phosphopeptides exhibiting significantly increased abundance levels with log2FC > 1 (Figure 4F) was performed to assess the biological variability between samples.

**Table 1:** List of Differentially Abundant Host Phosphoproteins with a Minimum Two-fold Increase in Phosphorylation in *M. bovis* BCG-WT Respective to the BCG-Δ*pknG* Mutant During Infection

UniProt ID	protein name	gene name	P-site	Log2FC
F6RJ39	Apoptotic chromatin condensation inducer in the nucleus	Acin1	S937; S656; S425	3.74; 2.47; 2.46
P25799	Nuclear factor NF-kappa-B p105 subunit	Nfkb1	S447	2.97
Q922U1	U4/U6 small nuclear ribonucleoprotein Prp3	Prpf3	T469	2.82
A2AU61	RNA-binding protein Raly	Raly	S119	2.48
Q9ERG0	LIM domain and actin-binding protein 1	Lima1	S735	2.27
Q9DBR1	5′-3′ exoribonuclease 2	Xrn2	S499; S501	1.84; 1.84
E9PZF0	Nucleoside diphosphate kinase A	Nme1	T94	1.81
O89053	Coronin-1A	Coro1a	T418	1.72
Q5EBP8	Heterogeneous nuclear ribonucleoprotein A1	Hnrnpa1	S6	1.68
P27546	Microtubule-associated protein 4	Map4	S517	1.64
H3BJU7	Rho guanine nucleotide exchange factor 2/GEF-H1	Arhgef2	S902	1.57
Q8C898	RIKEN cDNA C130026I21 gene	C130026I21Rik	S146	1.57
O35601	FYN-binding protein	Fyb	S561; Y559	1.55; 1.55
P14069	Protein S100-A6	S100a6	S46	1.53
Q9DBC7	cAMP-dependent protein kinase type I-alpha regulatory subunit	Prkar1a	S83	1.52
B9EJ86	Oxysterol-binding protein	Osbpl8	S314	1.49
E9PUI4	Protein-methionine sulfoxide oxidase MICAL1	Mical1	T895	1.48
Q9CYX7	RRP15-like protein	Rrp15	S265	1.40
E9PVX6	Proliferation marker protein *K*_i_-67	Mki67	T1159; S2392; S517	1.39; 1.26; 1.24
Q05D44	Eukaryotic translation initiation factor 5B	Eif5b	S215	1.36
Q8BUM3	Tyrosine-protein phosphatase nonreceptor type 7	Ptpn7	S93	1.34
E3UVT8	Anion exchange protein	Slc4a7	S1052; S233	1.30; 1.28
Q8CIH5	1-phosphatidylinositol 4,5-bisphosphate phosphodiesterase gamma-2	Plcg2	Y759	1.29
P84091	AP-2 complex subunit mu	Ap2m1	T156	1.29
Q9DBJ1	Phosphoglycerate mutase 1	Pgam1	S14	1.28
Q9CZW5	Mitochondrial import receptor subunit TOM70	Tomm70a	S94	1.28
A2A8V8	Serine/arginine repetitive matrix protein 1	Srrm1	S878	1.24
Q64337	Sequestosome-1	Sqstm1	T269; T272	1.24; 1.17
Q8JZQ9	Eukaryotic translation initiation factor 3 subunit B	Eif3b	S79	1.23
Z4YKC4	Eukaryotic translation initiation factor 4 gamma 3	Eif4g3	S274	1.20
Q99K01	Pyridoxal-dependent decarboxylase domain-containing protein 1	Pdxdc1	S737	1.16
F6WMJ3	Rho guanine nucleotide exchange factor 6	Arhgef6	S703	1.12
G3X9Q3	Minor histocompatibility protein HA-1	Hmha1	S568	1.11
Q5SQB0	Nucleophosmin	Npm1	S125	1.09
Q6P549	Phosphatidylinositol 3,4,5-trisphosphate 5-phosphatase 2	Inppl1	S132	1.07
O08583	THO complex subunit 4	Alyref	S237	1.06
B7ZCU2	Abl interactor 1	Abi1	S183	1.05
Q569Z6	Thyroid hormone receptor-associated protein 3	Thrap3	S248; S253; S243	1.04; 1.04; 1.02
Q61029	Lamina-associated polypeptide 2, isoforms beta/delta/epsilon/gamma	Tmpo	S183	1.04
Q60710	Deoxynucleoside triphosphate triphosphohydrolase SAMHD1	Samhd1	T52	1.01
Q8C3J5	Dedicator of cytokinesis protein 2	Dock2	S1729	1.01
E9Q2A6	Protein-tyrosine kinase 2-beta	Ptk2b	Y580	1.00

A presence/absence analysis identified phosphorylation events detected in at least 3/4 *M. bovis* BCG-WT replicates and absent in 4/4 BCG-Δ*pknG* mutant replicates. Thirty-four p-sites on 27 proteins were found exclusively in *M. bovis* BCG-WT (Tables 2 and S6). These phosphoproteins were also classified as having elevated abundance levels in the presence of PknG.

**Table 2:** List of Host Proteins Exclusively Phosphorylated in *M. bovis* BCG-WT

UniProt ID	protein name	gene name	P-site	localization probability
Q9JIY0	Pleckstrin homology domain-containing family O member 1	Plekho1	S270; T253	1.00; 1.00
Q9JHG6	Calcipressin-1	Rcan1	S161; S165	1.00; 1.00
Q6P5H2	Nestin	Nes	S1216; S813	1.00; 0.98
P55194	SH3 domain-binding protein 1	Sh3bp1	S677; S535	1.00; 0.89
A0A5F8MPZ2	Kinesin light chain	Klc1	S600	1.00
D3Z5N6	Zinc finger protein ubi-d4	Dpf2	S142	1.00
P18760	Cofilin-1	Cfl1	S41	1.00
P42932	T-complex protein 1 subunit theta	Cct8	S317	1.00
P46062	Signal-induced proliferation-associated protein 1	Sipa1	S8	1.00
Q01320	DNA topoisomerase 2-alpha	Top2a	S1521	1.00
Q8CIN4	Serine/threonine-protein kinase PAK 2	Pak2	S197	1.00
Q5SFM8	RNA-binding protein 27	Rbm27	T447	1.00
P50516	V-type proton ATPase catalytic subunit A	Atp6v1a	Y579	1.00
O70145	Neutrophil cytosol factor 2	Ncf2	S398	1.00
A0A1B0GR11	Transaldolase	Taldo1	S282	1.00
Q9EP97	Sentrin-specific protease 3	Senp3	S206	1.00
Q6PGL7	WASH complex subunit FAM21	Fam21	S533	1.00
A2ACV6	Protein SOGA1	Soga1	S1251	1.00
O35345	Importin subunit alpha 7	Kpna6	S6	1.00
Q9EQQ9	Protein O-GlcNAcase	Mgea5	S364	1.00
P97855	Ras GTPase-activating protein-binding protein 1	G3bp1	S149	0.99
A0A0R4J260	OTU domain-containing protein 4	Otud4	S999	0.99
Q9QXS1	Plectin	Plec	S4389	0.99
Q99K28	ADP-ribosylation factor GTPase-activating protein 2	Arfgap2	S145; S431	0.98; 0.90
Q3U1Z5	G-protein-signaling modulator 3	Gpsm3	S38	0.98
E9Q449	DENN domain-containing protein 4C	Dennd4c	S1323; S1297	0.97; 0.82
Q08024	Core-binding factor subunit beta	Cbfb	S173	0.97
Q61081	Hsp90 cochaperone Cdc37	Cdc37	S13	0.96
A0A3Q4EBK4	Myc box-dependent-interacting protein 1	Bin1	S267	0.95
Q8BVK9	Sp110 nuclear body protein	Sp110	S359	0.92
Q61033	Lamina-associated polypeptide 2, isoforms alpha/zeta	Tmpo	S308	0.89
A0A2I3BQE0	Serine/threonine-protein kinase 3	Stk3	T388	0.88
Q8CH18	Cell division cycle and apoptosis regulator protein 1	Ccar1	S453	0.85
Q8VDJ3	Vigilin	Hdlbp	S11	0.84
Q8C147	Dedicator of cytokinesis protein 8	Dock8	S905	0.80
A0A0A6YX02	Regulator complex protein LAMTOR1	Lamtor1	T28	0.77

### Functional Annotation of Differentially Abundant Host Proteins and Phosphoproteins in the Presence of PknG

A functional enrichment network was built for the significantly differentially abundant proteins and phosphoproteins by using the clusterProfiler package (Figure 5A,B). Enriched KEGG pathways of the differentially abundant proteins (Figure 5C) thereafter reaffirm PknG’s role in the modulation of host metabolism^32^ and the phagosome-lysosome interaction^9,11,33^ to control mycobacterial virulence. Among the functionally enriched KEGG pathways of the differentially phosphorylated proteins (Figure 5D), Fc gamma R-mediated phagocytosis, regulation of actin cytoskeleton, and endocytosis are consistent with previous literature surrounding the intracellular role of mycobacterial PknG during infection.^9,11,33^

**Figure 5:**
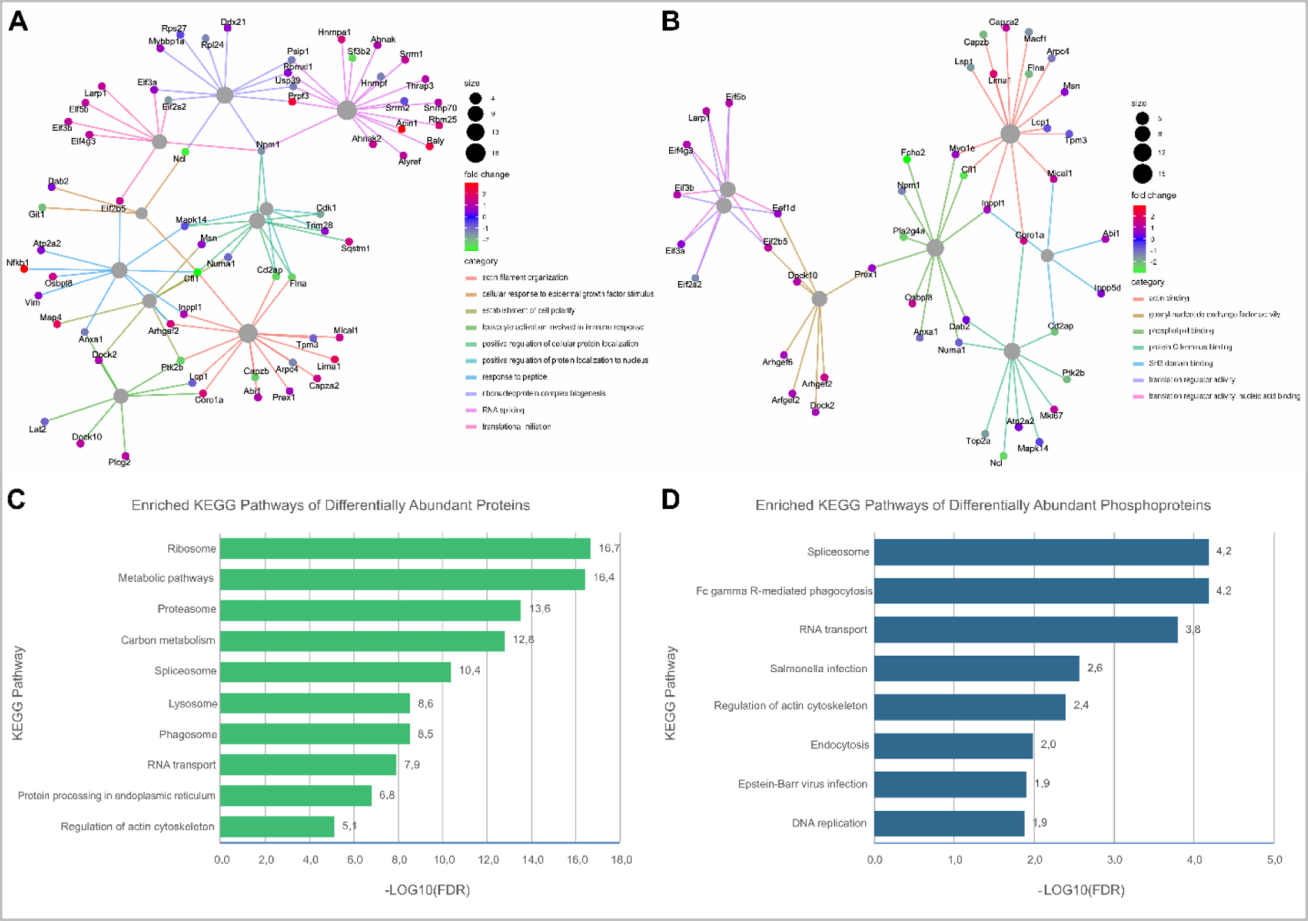
Visualization of functional and pathway enrichment analyses. Gene-concept network of differentially phosphorylated proteins in the presence of mycobacterial PknG associated with up to 10 most significant GO (A) biological processes and (B) molecular functions. Nodes are colored according to fold change and sized according to the number of associated proteins. Significantly enriched KEGG pathways of differentially abundant (C) proteins and (D) phosphoproteins. Bar charts ranking the significant KEGG pathways retrieved after functional enrichment analysis of the differentially abundant proteins and phosphoproteins in the presence of PknG during mycobacterial infection.

A cellular component GO enrichment analysis (Figure 6A) showed that cytoplasmic proteins were overrepresented among the proteins exhibiting an elevated phosphorylation level. Figure 6B shows a significant over-representation of translation initiation, actin cytoskeleton organization, apoptotic chromosome condensation, programmed cell death, and endosome organization pathways. Binding was observed as the most enriched molecular function among the differentially and uniquely phosphorylated proteins (Figure 6C) and describes molecule interactions dominated by GTPase binding, nucleic acid binding, and cytoskeletal protein binding. Moreover, we observed the enrichment of guanyl-nucleotide exchange factor (GEF) activity, which refers to the exchange of guanyl-nucleotides associated with a guanosine triphosphatase (GTPase).

**Figure 6:**
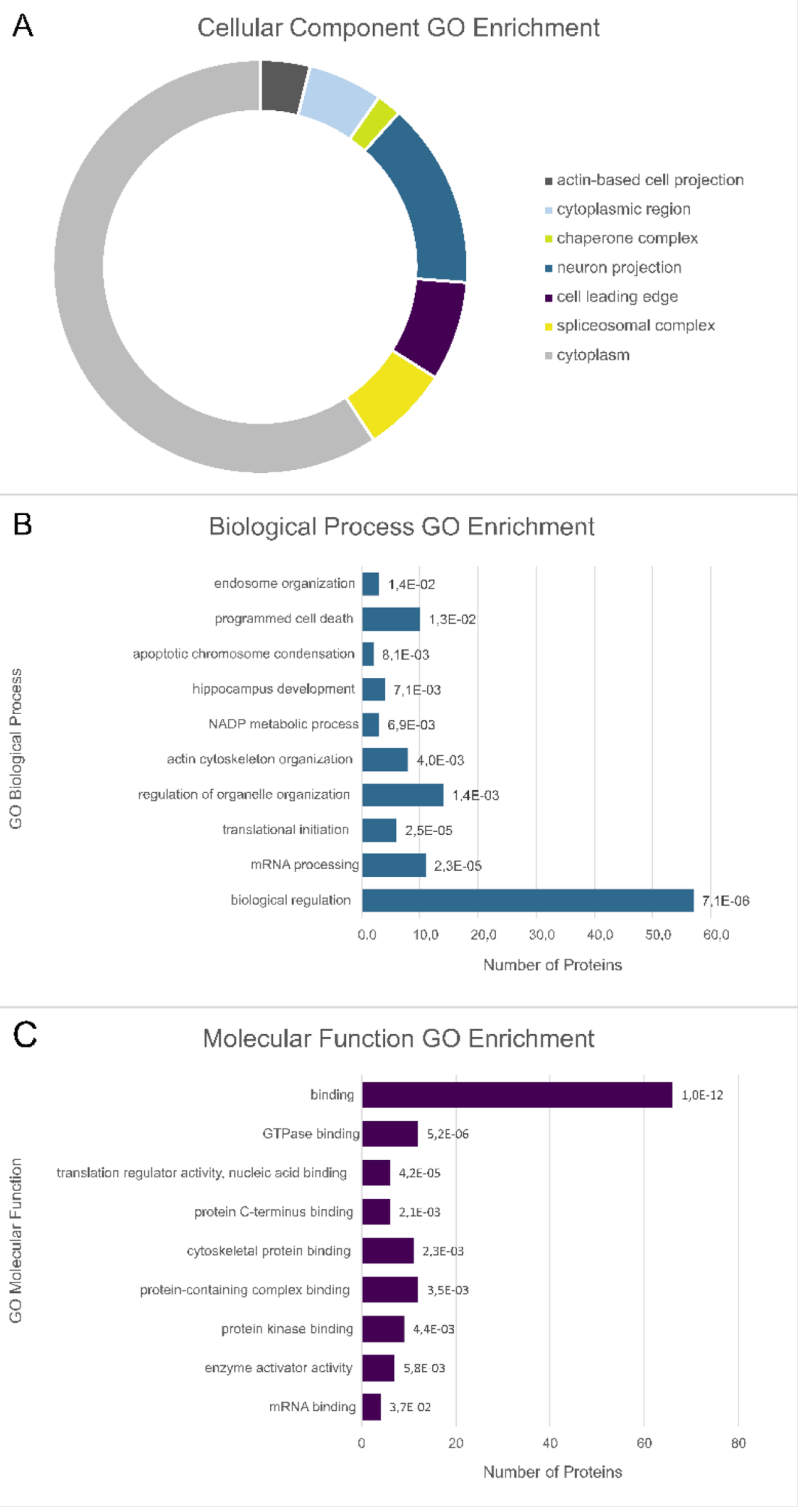
Significantly enriched GO terms associated with proteins exhibiting increased phosphorylation in the presence of mycobacterial PknG. (A) Pie chart illustrating the significantly enriched cellular component GO terms of the phosphoproteins with increased abundance. (B) Bar chart ranking of the significantly enriched biological process GO terms, and (C) molecular function GO terms of the phosphoproteins with increased abundance. Data labels represent FDR values for each enriched GO term.

### Identification of Enriched Sequence Motifs in Differentially Phosphorylated Substrates

To determine whether the PknG-responsive, differentially phosphorylated substrates (Tables 1 and 2) were enriched for specific phosphorylation motifs, relative to the broader host phosphoproteome, we used pLogo (https://plogo.uconn.edu/) for comparative visualization of over-represented amino acids flanking the phosphorylation sites on phosphopeptide sequence windows with increased abundance (Figure 7). Specifically, we compared phosphopeptides that were either significantly upregulated or exclusively detected in *M. bovis* BCG-WT-infected macrophages (“foreground”) against the entire observed phosphoproteome (“background”), and further compared the entire observed phosphoproteome against the *M. musculus* proteome.

**Figure 7:**
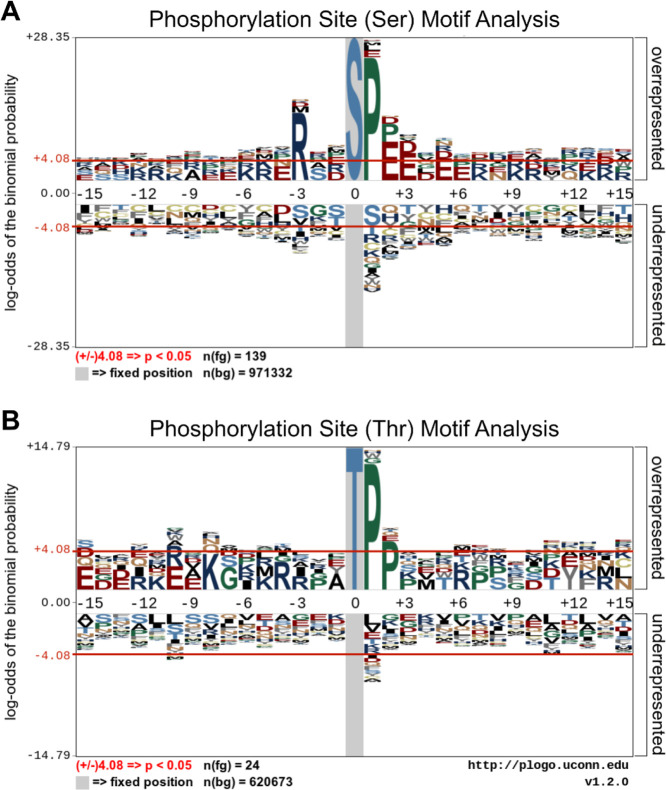
Phosphorylation site motif analyses of PknG-responsive phosphoproteins. (A) An in silico motif analysis of Ser-phosphorylated peptides (*n* = 139) derived from PknG-responsive phosphoproteins. (B) An in silico motif analysis of Thr-phosphorylated peptides (*n* = 24) derived from PknG-responsive phosphoproteins. Phosphorylation site motifs were analyzed using a foreground (fg) composed of available phosphopeptide sequence windows. The *Mus musculus* proteome was used as the background (bg) database. The red horizontal lines (±4.08) illustrate the relative statistical significance (*p*-value ≤ 0.05, after Bonferroni correction) of residues flanking the central phosphorylation site. Over-represented residues are above the midline, whereas under-represented residues are below the midline. Sequence logos were generated using pLogo.^20^

In the differential phosphoserine data set, significant enrichment was observed for tryptophan (W) at – 2, +2, and +5, and arginine (R) at +1. In the differential phospho-threonine data set, significant enrichment was observed for glutamine at +1 and arginine at – 15. However, no enrichment of classical SP, TP, or RXXS motifs was observed relative to background. By contrast, when we compared the global phosphoproteome against the mouse proteome, significantly enriched motifs include P at +1, R at −3, and acidic/proline-rich motifs (E at +2/+3, P at +2), consistent with known host kinase substrate preferences.

This motif analysis therefore suggests that the global phosphoproteome observed here is derived primarily from substrates of host kinases with known motifs (SP, TP, RXXS), as expected, while the PknG-responsive phosphoproteome likely largely reflects signal amplification through activation of host kinases in response to infection, rather than reflecting the direct substrate specificity of PknG per se. However, while the putative PknG-responsive phosphosites reported here are not enriched for SP, TP, or RXXS motifs, they are enriched for other motifs (vide supra), which may have upstream functional implications and warrants further investigation in future studies.

Based on these findings, we used the enriched motif classes, SP, TP, RXXS, and SXE as operational filters to identify specific host phosphoproteins containing these motifs and exhibiting significantly increased phosphorylation in BCG-WT-infected macrophages, which therefore likely represent downstream effectors rather than plausible direct targets of PknG (Table 3).

**Table 3:** List of PknG-Responsive Host Phosphoproteins Containing SP, TP, RXXS, or SXE Motifs

UniProt ID	protein name	gene name	P-site
F6RJ39	Apoptotic chromatin condensation inducer in the nucleus	Acin1	S937; S656; S425
E9Q616	AHNAK nucleoprotein (desmoyokin)	Ahnak	S4766; T496; T551; T4705
Q99K28	ADP-ribosylation factor GTPase-activating protein 2	Arfgap2	S145
A2A5R2	Brefeldin A-inhibited guanine nucleotide-exchange protein 2	Arfgef2	S227
Q60875	Rho guanine nucleotide exchange factor 2/GEF-H1	Arhgef2	S902
O55143	Sarcoplasmic/endoplasmic reticulum calcium ATPase 2	Atp2a2	S663
A0A3Q4EBK4	Bridging integrator 1	Bin1	S267
P47754	F-actin-capping protein subunit alpha-2	Capza2	S9
P42932	T-complex protein 1 subunit theta	Cct8	S317
O89053	Coronin-1A	Coro1a	T418
P98078	Disabled homologue 2	Dab2	T671
E9Q449	DENN domain-containing protein 4C	Dennd4c	S1323
Q8BZN6	Dedicator of cytokinesis protein 10	Dock10	S877
P57776	Elongation factor 1-delta	Eef1d	S133
Q99MS7	EH domain-binding protein 1-like protein 1	Ehbp1l1	S284
Q8CHW4	Translation initiation factor eIF-2B subunit epsilon	Eif2b5	S540
Q8BGD9	Eukaryotic translation initiation factor 4B	Eif4b	S406; S409
Z4YKC4	Eukaryotic translation initiation factor 4 gamma 3	Eif4g3	S274
P97855	Ras GTPase-activating protein-binding protein 1	G3bp1	S149
Q3THK7	GMP synthase [glutamine-hydrolyzing]	Gmps	S332
Q3TBD2	Minor histocompatibility protein HA-1/Rho GTPase-activating protein 45	Hmha1	S568
P49312	Heterogeneous nuclear ribonucleoprotein A1	Hnrnpa1	S6
O35345	Importin subunit alpha-7	Kpna6	S6
A0A0A6YX02	Ragulator complex protein LAMTOR1	Lamtor1	T28
Q6A0A2	La-related protein 4B	Larp4b	S603
P27546	Microtubule-associated protein 4	Map4	S517
P97310	DNA replication licensing factor MCM2	Mcm2	S21
E9PVX6	Proliferation marker protein *K*_i_-67	Mki67	S517; S2392; T1159
Q7TPV4	Myb-binding protein 1A	Mybbp1a	S1325
O70145	Neutrophil cytosol factor 2	Ncf2	S398
A0A0R4J260	OTU domain-containing protein 4	Otud4	S999
Q99K01	Pyridoxal-dependent decarboxylase domain-containing protein 1	Pdxdc1	S737
Q9DBC7	cAMP-dependent protein kinase type I-alpha regulatory subunit	Prkar1a	S77; S83
Q922U1	U4/U6 small nuclear ribonucleoprotein Prp3	Prpf3	T469
A2AU61	RNA-binding protein Raly	Raly	S119
Q8C2Q3	RNA-binding protein 14	Rbm14	S618
Q5SFM8	RNA-binding protein 27	Rbm27	T447
Q9JHG6	Calcipressin-1	Rcan1	S161; S165
F6YB25	Regulation of nuclear pre-mRNA domain-containing protein 1B	Rprd1b	S35
P14069	Protein S100-A6	S100a6	S46
P46062	Signal-induced proliferation-associated protein 1	Sipa1	S8
Q62376	U1 small nuclear ribonucleoprotein 70 kDa	Snrnp70	S226
Q8BVK9	Sp110 nuclear body protein	Sp110	S359
Q64337	Sequestosome-1	Sqstm1	T272; T269
Q569Z6	Thyroid hormone receptor-associated protein 3	Thrap3	S248; S253
Q61029	Lamina-associated polypeptide 2, isoforms beta/delta/epsilon/gamma	Tmpo	S183
Q9CZW5	Mitochondrial import receptor subunit TOM70	Tomm70a	S94
P83741	Serine/threonine-protein kinase WNK1	Wnk1	S2027

### Quantitative Analysis of Inflammatory Markers Modulated by PknG during Early Stage Infection

To comprehensively understand the early immunological and inflammatory profiles in response to the mycobacterial infection, the cell culture supernatants of macrophages were analyzed 30 min and 60 min postinfection using multiplexed immunoassays. The Olink Target 96 inflammation panel allowed for the simultaneous assessment of 92 protein biomarkers, presenting an expansive view of the inflammatory and immune responses triggered by both strains.

Notable immunological and inflammatory expression variations were identified between macrophages infected with the BCG-WT and BCG-Δ*pknG* strains (Tables 4 and 5), highlighting distinct immune response patterns triggered by each strain. At 30 min postinfection, the chemokine CCL3 showed significant downregulation in the WT-infected macrophages with a difference of −1.11 and a p-value of 1.14 × 10^–5^. Similarly, CX3CL1, CXCL1, and Flt3L also demonstrated down-regulated profiles in the WT-infected group.

**Table 4:** List of Differentially Regulated Inflammatory Biomarkers Post-30 min Infection Identified via PEA

assay	difference	*P*-value	regulation
CCL3	–1.11	1.14 × 10^–5^	Downregulated in WT
CDCP1	0.38	0.02	Upregulated in WT
CSF-1	–0.47	0.01	Downregulated in WT
CX3CL1	–0.50	0.02	Downregulated in WT
CXCL1	–0.61	0.04	Downregulated in WT
CXCL11	–0.34	0.02	Downregulated in WT
Flt3L	–0.96	0.03	Downregulated in WT

**Table 5:** List of Differentially Regulated Inflammatory Biomarkers Post-60 min Infection Identified via PEA

assay	difference	*P*-value	regulation
CXCL5	0.533	0.004	Upregulated in WT
FGF-5	0.325	0.006	Upregulated in WT
IL5	0.538	0.004	Upregulated in WT
MCP-3	0.433	0.012	Upregulated in WT
MMP-1	0.444	0.050	Upregulated in WT
NT-3	0.468	0.011	Upregulated in WT
OPG	0.339	0.003	Upregulated in WT
TNF	0.559	0.034	Upregulated in WT
TRAIL	0.405	0.017	Upregulated in WT
VEGFA	0.634	0.026	Upregulated in WT

In contrast, at 60 min postinfection with the WT strain, several inflammatory markers were upregulated. CXCL5, a recognized neutrophil attractant, was upregulated with a difference of 0.533 and a p-value of 0.004. Tumor necrosis factor (TNF), a principal proinflammatory cytokine, showed an upregulation with a difference of 0.559 and a p-value of 0.034. IL5, MMP-1, and FGF-5 also displayed elevated levels of expression in the WT-infected group, further highlighting the differential response. These differential expression profiles emphasize the distinct immune reactions triggered by the WT and BCG-Δ*pknG* strains of BCG.

## Discussion

The molecular basis of *Mtb*’s ability to avoid destruction following engulfment by host macrophages remains relatively poorly understood at the protein level. However, qualitatively, mycobacterial PknG—secreted via the accessory SecA2 pathway^36,37^ is thought to play an important role in reprogramming macrophage function to aid the survival of tubercle bacilli. *M. bovis* BCG, while attenuated in virulence, is pathogenic in immunosuppressed individuals and can cause TB-like disease. Moreover, *M. bovis* BCG shares >99% genomic similarity with *Mtb*, a phylogenetic distance of 0.1,^38^ and complete protein sequence identity for PknG, making it an effective model for studying how PknG may perturb host phosphorylation networks in infected macrophages. The functions of the differentially phosphorylated proteins identified in this study shed new light on the mechanisms that PknG uses to modulate and reprogram host-signaling pathways that enable the bacterium’s survival.

To explore the early phosphoproteomic responses to *M. bovis* BCG infection in macrophages, this study employed an MOI of 4 with a 30 min incubation and subsequent 30 min chase period. This setup effectively balanced bacillary internalization and macrophage viability, closely mirroring physiological infection conditions. Confocal and live-cell imaging provided clear evidence of mycobacterial internalization at these parameters, with confocal microscopy revealing internalized bacilli at 30 min postinfection. The proteomic analysis further confirmed this, identifying 25 *M. bovis* BCG proteins within macrophages, indicative of successful bacterial uptake. Moreover, CFU counts demonstrated the intracellular survival of BCG, with the WT strain showing a significant increase in CFU over 24 h, affirming the efficiency of our infection model.

The proteomic data set was normalized to a uniform quantity of extracted protein (550 μg), mitigating potential disparities in macrophage cell numbers at the 60 min postinfection mark. Consequently, the discernible upregulation of host proteins observed in WT infection cannot be ascribed to differences in cell viability. This normalization ensures the identified protein expression changes reflect genuine biological responses to infection rather than artifacts of varying cell counts.

### PknG-Induced Alterations in Host Sterol Metabolism and Mycobacterial Entry Mechanisms

At the proteomics level, a significant modulation in the abundance of key proteins involved in sterol metabolism was observed. The decreased abundance of NADH-cytochrome b5 reductase 1 (CYB5R1) and sterol O-acyltransferase 1 (SOAT1) in the presence of PknG indicates a potential disruption in the electron transport chain and cholesterol esterification process, respectively. Given the established dependency of mycobacteria on host-derived cholesterol for intracellular survival, these findings suggest a strategic bacterial interference in the host’s lipid metabolism, possibly to facilitate access to essential nutrients and create a favorable niche for its survival and replication.

### PknG Influences Cytoskeletal Organization and Phagosome Maturation by Modulating the Phosphorylation States of Host Proteins

Actin cytoskeletal rearrangement promotes numerous events beneficial to intracellular pathogens, including internalization of bacteria, altered vesicular trafficking, actin-dependent motility, and pathogen dissemination.^39^ Several groups have explored the role of the actin cytoskeleton during the *Mycobacterium* late phases of phagocytosis. Guérin & De Chastellier (2000) showed that *Mycobacterium avium* disrupts the macrophage actin filament network, highlighting the target for the bacterium that allows sustained intracellular survival.^40^ Anes et al. (2003) demonstrated that in contrast to nonpathogenic mycobacteria, pathogenic *Mtb* prevents actin polymerization on phagosomal membranes.^41^ The present study identified 20 proteins phosphorylated in the presence of PknG, which are known to contribute to cytoskeletal remodelling. This finding is consistent with a model in which PknG-responsive host protein phosphorylation is involved in the disruption of cytoskeleton remodelling.

The Rho family GTPases regulate the integrity of the actin cytoskeleton.^42^ GTPases RhoA, Rac1, and Cdc42 regulate crucial processes dependent on the actin cytoskeleton, such as cytokinesis, transcriptional activation, phagocytosis, morphology, and migration.^43–46^ The activation state of Rho GTPases is governed by the balance between the activities of GEFs and GTPase-activating proteins (GAPs).^47^

The differentially phosphorylated host protein GEF-H1 is a unique GEF that associates with microtubules and is known to mediate the crosstalk between microtubules and the actin cytoskeleton, which may contribute to the phagocytosis of bacteria by macrophages.^48^ Research has shown that GEF-H1 expression was increased via the MAPK signaling pathway during mycobacterial infection and silencing of GEF-H1 inhibited macrophage-mediated mycobacterial phagocytosis and elimination.^48^ Phosphorylation of GEF-H1 at S902 was identified, which presumably maintains GEF-H1 in a catalytically inactive configuration, thus inhibiting its GEF activity.^49,50^ This PknG-responsive, but likely indirect phosphorylation of GEF-H1 hinders RhoA activation, thereby contributing to the disruption/uncoupling of actin stress fibers and focal adhesions.

The Rac1/Cdc42 GEF, Rho guanine nucleotide exchange factor 6 (ARHGEF6) is required for the activation of Ser/Thr-protein kinase PAK 2 (PAK2) and subsequent LIM-kinase (LIMK)-dependent inactivation of cofilin. Cofilin-1 (CFL1) was significantly dephosphorylated in the presence of PknG in our data set. ARHGEF6 was found to be differentially phosphorylated at S663 and S703, and the phosphorylation of PAK2 at S2 and S197 showed increased abundance, likely having an inhibitory effect on ARHGEF6’s activation of PAK2. Collectively, the ARHGEF6/Rac1/PAK2/LIMK/cofilin signaling module limits actin turnover by confining both lamellipodial actin polymerization and depolymerization and promotes focal complex assembly in lymphocytes.^51^ Since phosphorylation of CFL1 suppresses its activity, our study indicates enhanced actin depolymerization and disruption of actin stress fibers and focal adhesion complexes in the presence of PknG. This potentially disrupts the fusion of phagosomes and lysosomes, a process dependent on actin polymerization. Moreover, our observation here of differential and unique phosphorylation of other GEFs and/or actin-binding proteins, including DENND4C, ARFGEF2, and DOCK10, further supports the hypothesis that PknG contributes to the blockade of phagosome-lysosome fusion by directly or indirectly modulating actin dynamics during infection.

The host F-actin-binding protein, coronin-1A [also known as tryptophan aspartate coat protein (TACO) or p57], was differentially phosphorylated at the p-site T418 in a PknG-responsive manner. It has been reported that phagosomes containing live pathogenic mycobacteria do not acquire the early endosomal protein marker Rab5 due to the transient recruitment and active retention of coronin-1A, thereby inhibiting phagosomal maturation.^52^ Notably, the phosphorylation of coronin-1A deregulates its association with F-actin, which, in turn, facilitates early phagosome formation. Increased phosphorylation of coronin-1A at T418 was also previously observed during the first 10 min after lipopolysaccharide (LPS) and Pam3Cys stimulation of macrophages.^53^ Together, our results suggest that PknG-responsive phosphorylation of GEFs, GAPs, and actin-binding proteins aids pathogenic mycobacteria to establish its niche within host macrophages through the disruption of actin stress fibers, actin depolymerization, and inhibition of phagosome maturation.

### PknG Manipulates Host Programmed Cell Death and Inflammatory Pathways to Facilitate Mycobacterial Survival

*Mtb* infection triggers intracellular signaling pathways, enhancing pro-inflammatory responses that are crucial for controlling *Mtb* replication and the immunopathologic response.^54^ Autophagy contributes to killing intracellular microbes, including *Mtb*, by modulating host resistance against infections and controlling cellular survival.^55–57^ Autophagy activation also aids in regulating inflammation, contributing to a more efficient innate immune response against *Mtb*. In vitro studies have shown that mycobacteria escaping from phagosomes into the cytosol are ubiquitinated and targeted by selective autophagy receptors,^58,59^ such as Sequestosome 1 (SQSTM1, also known as p62).

Herein, SQSTM1 was identified as differentially phosphorylated at residues T269 and T272 in a PknG-responsive manner. This signaling adaptor is central to cell survival and proliferation through the activation of the mechanistic target of rapamycin complex 1 (mTORC1), which facilitates autophagy inhibition.^60^ Phosphorylation of T269 and S272 (T272 in *M. musculus*) on SQSTM1 has been reported to be necessary for autophagic inhibition under nutrient-rich conditions.^61^ During impaired autophagy, SQSTM1 accumulates and activates inflammation via nuclear factor kappa B (NF-κB).^62^ NF-κB1 subunit p105 (NF-κB1) was differentially phosphorylated at S447. NF-κB1 functions both as a precursor of NF-κB p50 and as a cytoplasmic inhibitor of NF-κB. The consequence(s) of S447 phosphorylation have yet to be explored. However, well-studied p150 p-sites, such as S893, S903, S907, S927, and S932, point to phosphorylation being a critical factor in the outcome of proteasomal processing (partial or complete degradation) of p105.^63–65^

Importantly, p105 is also a negative regulator of MAPK activation downstream of various receptors, including TLRs and TNF receptors.^66,67^ The mycobacterial cell wall contains several pro-inflammatory TLR2 ligands and induces activation of the MAPKs and NF-κB pathways.^68,69^ The MAPK pathways play an important role in enhancing antimycobacterial activity and the production of pro-inflammatory mediators, including TNF-α,^68^ which contribute to phagocytosis, intracellular killing, T cell activation, and granuloma formation.^70^ Wu et al. (2018) demonstrated that overexpression of PknG in *M. smegmatis* *::*PknG infected THP1 macrophages resulted in decreased intracellular cytokine levels, thus promoting mycobacterial survival.^71^ In particular, PknG was shown by Wu et al. to inhibit the inflammatory response by suppressing NF-κB and ERK1/2 pathways, albeit no phosphoproteomic analysis was reported in that study. Hence, our findings of differential phosphorylation of NF-κB1 p105 potentially provide a mechanistic basis for the inhibitory role of PknG in pro-inflammatory cytokine induction.

Similarly, apoptosis of infected macrophages is associated with diminished pathogen viability.^72^ However, virulent mycobacteria are known to evade apoptosis, which aids in the pathogenesis of these strains.^73,74^ Apoptotic chromatin condensation inducer in the nucleus (ACIN1) is a multifunctional protein with proposed roles in apoptosis and alternative RNA splicing.^75^ We identified ACIN1 as differentially phosphorylated at sites S937, S656, and S425 in a PknG-responsive manner. Acin1 undergoes several proteolytic cleavages during apoptosis. However, Akt-mediated phosphorylation of ACIN1 on S422 and S573 residues promotes resistance against proteolytic/apoptotic cleavage in the nucleus and inhibits ACIN1-dependent chromatin condensation, thereby facilitating cell survival.^76^ Interestingly, Banfalvi (2014) identified anomalies in chromatin condensation associated with the apoptosis process in *Mtb*-infected macrophages.^77^

ACIN1 is also an auxiliary component of the exon junction complex (EJC) assembled during pre-mRNA splicing. The recruitment of a trimeric complex composed of ACIN1, SAP18, and RNPS1 to the EJC was reported to modulate the apoptotic and splicing regulation. Nevertheless, ACIN1 independently regulates the splicing profiles of apoptotic genes and retinoic acid-induced splicing events.^78,79^ The data presented here thus suggest that PknG plays an indirect role in inhibiting apoptosis to promote bacterial survival and persistence of pathogenic mycobacteria.

### Distinct Two-Phased Immune Evasion and Activation Dynamics

Our Olink PEA analysis revealed a distinct two-phase immune response in RAW 264.7 macrophages post-BCG-WT infection. Initially, there was a marked suppression of chemokines CCL3, CX3CL1, CXCL1, and CXCL11, which are instrumental in immune cell recruitment, indicating an early immune response inhibition strategy by the WT strain. This was accompanied by reduced levels of CSF-1 and Flt3L, potentially indicating compromised macrophage and dendritic cell activity. Subsequently, after 60 min, cytokines such as CXCL5 and TNF were upregulated, indicative of a heightened immune response. This two-phase immune response, characterized by initial suppression followed by robust inflammation, suggests a survival strategy of the WT strain to both establish and sustain infection, with PknG playing a central role in this immune modulation.

## Conclusions

This study utilized label-free MS-based phosphoproteomics to elucidate the complex mechanisms employed by pathogenic mycobacteria, particularly those mediated by PknG, to modulate host macrophage functions and ensure its survival postphagocytosis. Our findings reveal several diverse and intricate mechanisms by which the presence of PknG perturbs host cell processes, including cytoskeletal organization, phagosome maturation, sterol metabolism, and programmed cell death pathways. While existing literature corroborates these perturbations, our phosphoproteomic data have offered novel, in-depth mechanistic perspectives on the post-translational regulation of these host processes, where PknG appears to play a significant, albeit not exclusive, role.

Affinity proteomic data on the secreted proteome indicated a dual-phase modulation of the immune response in macrophages upon infection with *M. bovis* BCG, highlighting a strategic bacterial tactic to initially suppress and subsequently stimulate host immune responses. This biphasic pattern underscores a sophisticated microbial survival strategy facilitated by PknG to establish and sustain infection within the host. Our proteomic data further demonstrate PknG’s extensive impact on host sterol metabolism and actin cytoskeletal dynamics. Thus, this work considerably augments the current understanding of mycobacterial pathogenesis and provides hypotheses for the development of new molecular strategies aimed at reactivating specific host cellular pathways to restore the macrophage capability of mycobacterial elimination.

A limitation of this study arises from the inherent challenges in analyzing the protein complexity and dynamic range of (phospho)­protein concentrations in infected macrophages. Given the dynamic range constraints of the Q Exactive mass spectrometer used to identify and quantify individual tryptic (phospho)­peptides, our data set likely omits proteins at the lower end of the abundance range. Although our data did not (and was not expected to) directly detect PknG and, therefore, could not conclusively prove its escape from phagosomal localization in our infection model, the evidence presented here and elsewhere^82^ strongly suggests that this does happen, presenting a testable hypothesis for future research. Additionally, our experimental design does not account for potential bystander effects, such as nonphosphorylation-mediated protein–protein interactions. Ongoing and future studies, including mycobacterial infections in alveolar macrophage cell lines and primary cells, aim to validate and expand upon these findings.

This study does not provide biochemical confirmation of direct phosphorylation by PknG for the list of candidate host substrates. In vitro kinase assays or ectopic overexpression of PknG were not performed due to perceived limitations in physiological relevance and the potential for altered localization or cofactor availability. Indeed, Saha et al. (2025) recently reported that recombinant PknG failed to phosphorylate SODD in vitro unless PknG was first S-nitrosylated, highlighting the complexity of kinase activity under physiological conditions.^82^ The differential phosphoproteomic comparison between BCG-WT and Δ*pknG* infections identifies phosphorylation events that depend on the presence of PknG, but these events may arise from direct phosphorylation or indirectly through modulation of host kinase and phosphatase activity.

## Supplementary Material





















## Data Availability

The MS proteomics and phosphoproteomics data have been deposited to the ProteomeXchange Consortium (http://proteomecentral.proteomexchange.org) via the PRIDE partner repository with the data set identifier PXD031055

## Notes

Relative fractions of pS, pT and pY observed in the phosphoproteome data set; Stacked bar plot illustrating the relative fractions of pS, pT, and pY among the differential and nondifferential p-site, peptide, and protein identifications; list of high confidence *M. bovis* BCG protein identifications; list of differentially abundant host proteins in *M. bovis* BCG-WT respective to the BCG-Δ*pknG* mutant during RAW 264.7 macrophage infection; list of differentially up-regulated host proteins in *M. bovis* BCG-WT respective to the BCG-Δ*pknG* mutant during RAW 264.7 macrophage infection; list of differentially phosphorylated host peptides in *M. bovis* BCG-WT respective to the BCG-Δ*pknG* mutant during RAW 264.7 macrophage infection; list of differentially up-regulated host phosphopeptides in *M. bovis* BCG-WT respective to the BCG-Δ*pknG* knockout mutant during RAW 264.7 macrophage infection; list of host proteins exclusively phosphorylated in RAW 264.7 macrophages infected with *M. bovis* BCG-WT; field of view 1—time-lapse live-cell imaging of *M. bovis* BCG internalization by macrophages; field of view 2—time-lapse live-cell imaging of *M. bovis* BCG internalization by macrophages; field of view 3—time-lapse live-cell imaging of *M. bovis* BCG internalization by macrophages; field of view 4—time-lapse live-cell imaging of *M. bovis* BCG internalization by macrophages; field of view 5—time-lapse live-cell imaging of *M. bovis* BCG internalization by macrophages; field of view 6—time-lapse live-cell imaging of *M. bovis* BCG internalization by macrophages; field of view 7—time-lapse live-cell imaging of *M. bovis* BCG internalization by macrophages; and field of view 8—time-lapse live-cell imaging of *M. bovis* BCG internalization by macrophages (PDF)
Table S1. List of high confidence *M. bovis* BCG protein identifications. Table S2. List of differentially abundant host proteins in *M. bovis* BCG-WT respective to the BCG-Δ*pknG* mutant during RAW 264.7 macrophage infection. Table S3. List of differentially up-regulated host proteins in *M. bovis* BCG-WT respective to the BCG-Δ*pknG* mutant during RAW 264.7 macrophage infection. Table S4. List of differentially phosphorylated host peptides in *M. bovis* BCG-WT respective to the BCG-Δ*pknG* mutant during RAW 264.7 macrophage infection. Table S5. List of differentially up-regulated host phosphopeptides in *M. bovis* BCG-WT respective to the BCG-Δ*pknG* knock-out mutant during RAW 264.7 macrophage infection. Table S6. List of host proteins exclusively phosphorylated in RAW 264.7 macrophages infected with *M. bovis* BCG-WT. Table S7. List of candidate host substrates of PknG with sequence windows. Table S8. Pairs of receptor/peptide residues closer than 4.5 Å in the selected complex (XLSX)
Video S1. Field of view 1 - Time-lapse live-cell imaging of *M. bovis* BCG internalisation by macrophages (AVI)
Video S2. Field of view 2 - Time-lapse live-cell imaging of *M. bovis* BCG internalisation by macrophages (AVI)
Video S3. Field of view 3 - Time-lapse live-cell imaging of *M. bovis* BCG internalisation by macrophages (AVI)
Video S4. Field of view 4 - Time-lapse live-cell imaging of *M. bovis* BCG internalisation by macrophages (AVI)
Video S5. Field of view 5 - Time-lapse live-cell imaging of *M. bovis* BCG internalisation by macrophages (AVI)
Video S6. Field of view 6 - Time-lapse live-cell imaging of *M. bovis* BCG internalisation by macrophages (AVI)
Video S7. Field of view 7 - Time-lapse live-cell imaging of *M. bovis* BCG internalisation by macrophages (AVI)
Video S8. Field of view 8 - Time-lapse live-cell imaging of *M. bovis* BCG internalisation by macrophages (AVI)
