# Supplementary material for: A Phosphoproteomic Analysis of Mycobacterial PknG-Mediated Host Immune Evasion
Source: J Proteome Res. 2025 Oct 9;24(11):5585–603. doi: 10.1021/acs.jproteome.5c00416 (PMC12604046; doi:10.1021/acs.jproteome.5c00416)
Supplement: Supplementary file 1 [file pr5c00416_si_001.pdf]

A Phosphoproteomic Analysis of Mycobacterial PknG-Mediated Host Immune Evasion

Seanantha S. Baros-Steyl[1], Kehilwe C. Nakedi[1], Tariq A. Ganief[1,2], Nelson C. Soares[3,4,5,6]* and Jonathan M. Blackburn[1,2]*

[1]Division of Chemical & Systems Biology, Department of Integrative Biomedical Sciences, Faculty of Health Sciences, University of Cape Town, Cape Town 7925, South Africa.

[2]Institute of Infectious Disease & Molecular Medicine, Faculty of Health Sciences, University of Cape Town, Cape Town 7925, South Africa.

[3]Center for Applied Translation Genomics, Mohammed Bin Rashid University of Medicine and Health Sciences, Dubai Health, Dubai 505055, United Emirates.

[4]College of Medicine, Mohammed Bin Rashid University of Medicine and Health Sciences, Dubai Health, Dubai 505055, United Arab Emirates.

[5]Laboratory of Proteomics, Department of Human Genetics, National Institute of Health Doutor Ricardo Jorge (INSA), Lisbon 1649-016, Portugal.

[6]Comprehensive Health Research Centre (CHRC), NOVA Medical School, University NOVA of Lisbon, Lisbon 1169-056, Portugal.

Supporting Information - Table of Contents

Figure S1. Relative fractions of pS, pT and pY observed in the phosphoproteome dataset.

Table S1. List of high confidence M. *bovis* BCG protein identifications.

Table S2. List of differentially abundant host proteins in M. *bovis* BCG-WT respective to the BCG-Δ*pknG* mutant during RAW 264.7 macrophage infection.

Table S3. List of differentially up-regulated host proteins in M. *bovis* BCG-WT respective to the BCG-Δ*pknG* mutant during RAW 264.7 macrophage infection.

Table S4. List of differentially phosphorylated host peptides in M. *bovis* BCG-WT respective to the BCG-Δ*pknG* mutant during RAW 264.7 macrophage infection.

Table S5. List of differentially up-regulated host phosphopeptides in M. *bovis* BCG-WT respective to the BCG-Δ*pknG* knock-out mutant during RAW 264.7 macrophage infection.

Table S6. List of host proteins exclusively phosphorylated in RAW 264.7 macrophages infected with M. *bovis* BCG-WT.

Video S1. Field of view 1 - Time-lapse live-cell imaging of M. *bovis* BCG internalisation by macrophages.

Video S2. Field of view 2 - Time-lapse live-cell imaging of M. *bovis* BCG internalisation by macrophages.

Video S3. Field of view 3 - Time-lapse live-cell imaging of M. *bovis* BCG internalisation by macrophages.

Video S4. Field of view 4 - Time-lapse live-cell imaging of M. *bovis* BCG internalisation by macrophages.

Video S5. Field of view 5 - Time-lapse live-cell imaging of M. *bovis* BCG internalisation by macrophages.

Video S6. Field of view 6 - Time-lapse live-cell imaging of M. *bovis* BCG internalisation by macrophages.

Video S7. Field of view 7 - Time-lapse live-cell imaging of M. *bovis* BCG internalisation by macrophages.

Video S8. Field of view 8 - Time-lapse live-cell imaging of M. *bovis* BCG internalisation by macrophages.

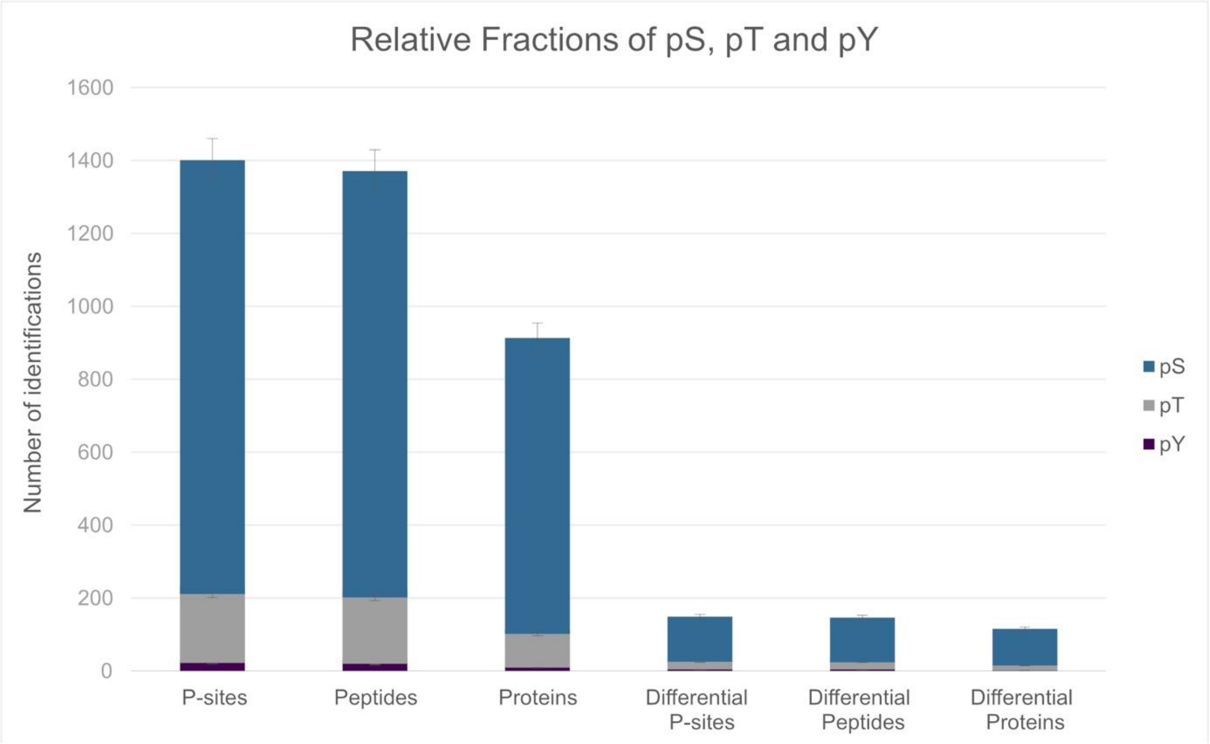

Figure S1. Relative fractions of pS, pT and pY observed in the phosphoproteome dataset.
